# Breathlessness in the general population

**DOI:** 10.1097/SPC.0000000000000751

**Published:** 2025-03-05

**Authors:** Alexander Müller, Emiel F.M. Wouters, Daisy J.A. Janssen

**Affiliations:** a Ludwig Boltzmann Institute for Lung Health, Vienna, Austria; b Department of Health Services Research, Care and Public Health Research Institute, Faculty of Health, Medicine and Life sciences, Maastricht University, Maastricht, Netherlands; c Faculty of Medicine, Sigmund Freud Private University, Vienna, Austria; d Department of Respiratory Medicine, Maastricht University Medical Center, Maastricht, The Netherlands; e Department of Family Medicine, Care and Public Health Research Institute, Faculty of Health, Medicine and Life sciences, Maastricht University, Maastricht, Netherlands; f Department of Expertise and treatment, Proteion, Horn, the Netherlands

**Keywords:** breathlessness, dyspnoea, general population

## Abstract

**Purpose of the review:**

Breathlessness is a prevalent symptom that significantly affects physical and mental health. While commonly associated with respiratory and cardiovascular diseases, breathlessness is increasingly recognised as a concern in the general population. This review summarises recent research on the prevalence, risk factors, assessment methods, and clinical and societal impact, with a focus on findings from the past 18 months.

**Recent findings:**

Recent studies indicate that breathlessness affects a substantial proportion of adults worldwide, with prevalence varying across populations and regions. Identified risk factors include older age, female sex, high body mass index, smoking, and comorbidities such as respiratory and cardiovascular diseases. Novel approaches in assessing breathlessness are looking beyond unidimensional scales to improve diagnostic accuracy. However, breathlessness remains underdiagnosed in clinical practice. Recent publications also show that breathlessness has a substantial impact on health outcomes of the affected person, but also imposes a burden on their informal caregivers, health care systems and the economy.

**Summary:**

Despite progress in understanding chronic breathlessness, knowledge gaps persist, particularly regarding its assessment in large-population samples. Longitudinal studies are needed to understand risk factors for breathlessness and its impact on health outcomes and society.

## INTRODUCTION

Breathlessness, or dyspnoea, is a complex phenomenon of breathing discomfort and increased work of breathing that significantly impacts physical and mental dimensions of health [1]. Breathlessness is commonly associated with acute and chronic conditions such as chronic obstructive pulmonary disease (COPD), asthma, and heart failure. However, there is a growing recognition of breathlessness as a global health concern. Moreover, in low- and middle-income countries (LMICs), its burden is likely underreported and strongly influenced by environmental and socioeconomic factors [2].

In 2022, the World Health Organization (WHO) included ‘chronic breathlessness’ in the International Classification of Diseases (ICD-11) [3]. Chronic breathlessness is defined as breathlessness being present for more than eight weeks. Chronic breathlessness often persists despite optimal treatment of underlying diseases, requiring introducing (palliative) symptom management alongside treatment focusing on disease modification [3].

Recent advances in research have deepened our understanding of the prevalence of breathlessness, its underlying factors, and the broader implications for clinical practice and health care systems. This narrative review summarises recent publications to illuminate the current state of research on breathlessness in the general population. We will thereby focus on the prevalence and epidemiological characteristics of (chronic) breathlessness, its assessment as well as its clinical impact. Finally, we will identify areas for future research.

**KEY POINTS**

- There is a growing interest on breathlessness as a symptom in the general population.
- Chronic breathlessness in the general population is strongly associated with overweight, older age, female sex and low lung function among other factors.
- Chronic breathlessness imposes a substantial burden to individuals affected by it, their informal caregivers and society.

## PREVALENCE AND EPIDEMIOLOGICAL CHARACTERISTICS OF BREATHLESSNESS

A 2023 systematic review showed that breathlessness is experienced by approximately 10% of adults in high-income countries, with prevalence rates rising with age and in association with comorbidities such as obesity, respiratory and cardiovascular disease [4]. The review further highlighted that the modified Medical Research Council (mMRC) breathlessness scale is the most widely used tool for assessing breathlessness in population-based studies.

Recently published studies on prevalence and epidemiological characteristics of chronic breathlessness help in understanding this symptom from a more global perspective. This is important, as most of the previous data on this topic were derived from Europe, North America or Australia [4]. Findings from the multinational Burden of Obstructive Lung Disease study show that the prevalence of clinically relevant breathlessness (mMRC ≥2) averages 13.7% across the 41 study sites, with substantial geographical variation, ranging from 0% in a site in India to 28.8% in a site in the Philippines [5]. This study comprised sites from 5 continents including many from LMICs. The strong variation of breathlessness prevalence could not be explained by geographical region or national income but might be partly caused by cultural or language-based differences in reporting subjective symptoms. The study also identified a strong association between breathlessness and reduced lung function.

A recent publication by Kochovska *et al.* based on a population-based online survey among 3046 participants in India, found that 44% of adults reported some degree of breathlessness on exertion (mMRC ≥1), with 17% attributing it to lung conditions, 13% to anaemia, and 28% to poor nutrition [6]. The authors concluded that the high prevalence of exertional breathlessness indicates a relevant public health concern, as this affects more than 600 million people in India alone. Nevertheless, it must be highlighted, that the authors of this study used a score of 1 or higher on the mMRC breathlessness scale to define clinically relevant breathlessness while most previous publications used a cut-off score of 2, thus explaining the very high prevalence estimate for breathlessness compared to other studies.

In middle-aged adults, the Swedish CArdioPulmonary bioImage Study (SCAPIS) reported a breathlessness prevalence of 3.7% (mMRC ≥2), with the strongest contributing factors being overweight and obesity (population attributable fraction [PAF] 59.6–66.0%), stress (PAF 31.6–76.8%), respiratory disease (PAF 20.1–37.1%), and depression (PAF 17.1–26.6%) [7]. Another publication using the same dataset and applying a machine-learning algorithm to identify factors related to breathlessness also found forced expiratory volume in 1 second (FEV1) and physical activity to be relevant parameters beside overweight and obesity [8].

**FIGURE 1.**
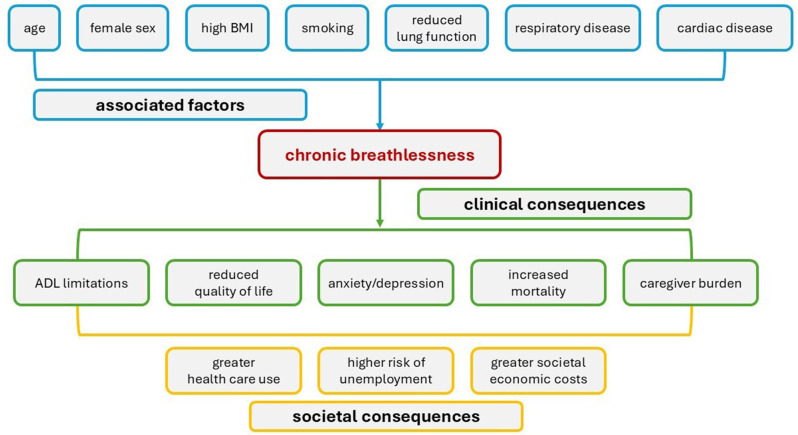
Most reported associated factors, clinical and societal consequences of chronic breathlessness. Abbreviations: BMI = body mass index, ADL = activities of daily living.

Summarising these recently published studies, several demographic and clinical factors seem to be associated with breathlessness: Across studies, older age, female sex, and smoking history are consistently identified as risk factors (see Fig. 1). Being overweight and obese also plays a crucial role, especially in exertional breathlessness, with increased body mass index (BMI) being one of the strongest predictors of breathlessness in population-based studies. In addition to these factors, respiratory diseases such as COPD and asthma, as well as cardiovascular conditions, anaemia, and reduced lung function, have been implicated in the symptom’s pathophysiology. The presence of multiple comorbidities further seems to exacerbate breathlessness, highlighting the multifactorial causes of chronic breathlessness. The variation in prevalence across studies suggests that environmental, socioeconomic, and health care access factors may also contribute to disparities in symptom burden, although these assumptions cannot be confirmed by the currently available data. One major limitation that persists in epidemiological research on breathlessness is the lack of longitudinal data investigating changes in prevalence estimates as well as causes for breathlessness in the general population. Currently available cross-sectional analyses can show correlations but cannot study causation and therefore lack identifying relevant risk factors leading to the development of breathlessness. Another limitation is imposed by the varying definitions of breathlessness in general population studies by using different scales and cut-off values and thus reducing comparability of results. In addition, most research does not cover chronic breathlessness based on the current ICD-11 definition, as the duration of symptoms for more than 8 weeks is not assessed.

## ASSESSMENT OF BREATHLESSNESS

The mMRC scale remains the most widely used tool for assessing breathlessness in population-based studies [4]. The mMRC scale provides a simple, graded classification of breathlessness severity based on functional limitations, making it a feasible and easy to administer choice for epidemiological research. It was designed and validated for the use in specific populations such as patients with COPD, whereas its application in general population studies is questionable. The mMRC scale relies on subjective self-reporting. Its unidimensional design and its limited ability to capture fluctuations in breathlessness across different contexts and exertion levels call for the development of more nuanced assessment approaches.

Recent advancements in breathlessness assessment have focused on refining measurement tools to improve clinical and research applicability. Tools like the Multidimensional Dyspnoea Profile (MDP) allow for a more holistic approach in assessing clinically relevant breathlessness while still using subjective scales and thus being easy to apply [9]. In addition, the MDP is validated for the use in different timeframes and conditions and can therefore also assess chronicity of symptoms in population-based studies.

Beyond subjective ratings, physiological and performance-based measures are increasingly being integrated into breathlessness assessment, not only in clinical, but also in epidemiological research. Ekström *et al.* proposed the use of normative reference equations to standardise breathlessness intensity evaluation during exertion [10▪▪,^11^]. These reference equations predict expected breathlessness responses based on individual characteristics (such as age, sex, height, and body mass) and level of exertion (e.g., distance walked in a six-minute walk test or power output during an incremental cycle cardiopulmonary exercise test). The authors thereby built on a preceding study derived from a clinical setting [12] and expanded the findings to population-based research. By contextualising breathlessness within exertional capacity, these methods provide a more precise differentiation between clinically relevant pathological breathlessness and normal exertional breathlessness. This approach represents a significant advance over traditional self-reported scales, allowing for more individualised assessments and potentially improving clinical decision-making. A recently published analysis in 318 COPD patients showed that mMRC was not sensitive enough to validly identify abnormal exertional breathlessness as measured by Borg scale ratings during cardiopulmonary exercise testing [13]. Although these results were not performed in a large population-based setting, they underline the potential need for objective measurements in addition to subjective scales to identify breathlessness. Furthermore, these new approaches of combining subjectively reported outcomes with objectively measurable parameters, might help in comparing epidemiological data on breathlessness across world regions, as it might reduce the previously described culture and language bias.

These advancements underscore a shift from a focus on the mMRC scale toward multidimensional and exertion-calibrated breathlessness assessment. While the mMRC scale is easily applicable for large-scale epidemiological studies, emerging methods might be more precise in assessing breathlessness in this context. However, the feasibility of these more refined assessment tools in large samples must be further investigated.

## THE CLINICAL IMPACT OF BREATHLESSNESS

Breathlessness is a prevalent and distressing symptom that significantly affects individuals’ daily lives, yet it often remains under recognised in clinical practice. A recent national online survey conducted by Kochovska *et al.* found that among individuals reporting breathlessness, nearly 70% had initiated discussions about their symptom with their health care provider, whereas in 30% of cases, breathlessness remained unaddressed during consultations [14]. Notably, for those with severe breathlessness (mMRC 3–4), 24% reported that the symptom was never discussed in their medical visits, despite its significant impact on their well-being. This highlights a critical gap in clinical history-taking and symptom management, suggesting that breathlessness is frequently ‘invisible’ in consultations unless explicitly raised by patients. Identifying breathlessness in an early stage is relevant, as the clinical consequences of breathlessness for individuals affected by it are diverse (see Fig. 1).

### The association of breathlessness and other symptoms

Breathlessness rarely occurs in isolation and is often associated with other debilitating symptoms, including fatigue, anxiety, and depression. A moderate relation between breathlessness and fatigue was previously reported in asthma patients [15]. Findings from the recently published VAScular disease and Chronic Obstructive Lung disease study in older men indicate that breathlessness was also strongly linked to fatigue in this cohort [16]. Using the MDP and the Dyspnoea-12 questionnaire, the authors showed that some dimensions of breathlessness, such as overall intensity and unpleasantness, were found to have a direct correlation with increased fatigue severity, independent of confounding factors such as cardiovascular disease and respiratory conditions. This also suggests that multidimensional assessment tools may be more appropriate in capturing the complex interplay between breathlessness and associated symptoms. Moreover, breathlessness has been linked to heightened levels of anxiety and depression, further compounding its impact on individuals’ overall health status [6,17].

### Impact of breathlessness on physical disability and quality of life

Breathlessness profoundly affects quality of life, limits mobility, social participation, and overall well-being. A cross-sectional study by Kochovska *et al.* revealed that individuals with persistent breathlessness reported significantly poorer perceived health compared to those without the symptom [17]. In particular, worsening breathlessness was strongly correlated with worsening self-rated health, increased physical limitations, and higher levels of psychological distress. Similar findings were also presented in the previously mentioned large population-base study in Indian adults. In this study, the mMRC score was strongly associated with both the World Health Organization Disability Assessment Schedule (WHODAS-12) score for disability and the European Quality of Life 5 Dimensions 5 Level Version (EQ-5D-5L) scores for quality of life [6]. Furthermore, another population-based study found that individuals experiencing chronic breathlessness often reduce their daily activities to manage their symptoms, leading to progressive deconditioning and further exacerbation of functional impairment [18]. Again, all these studies show associations based on cross-sectional data only. Studies investigating the impact of breathlessness on disability and quality of life over a longer period are currently lacking. Longitudinal analyses on this topic should be a priority for future research.

### Breathlessness as a predictor of morbidity and mortality

The prognostic significance of breathlessness has been increasingly recognised in clinical research. A retrospective cohort study in an adult Swedish population-based sample demonstrated that abnormally high exertional breathlessness during incremental exercise testing was a strong predictor of all-cause, respiratory, and cardiac mortality [19]. Even in individuals with normal exercise capacity, heightened breathlessness responses were associated with a 50% increased risk of all-cause mortality compared to those with normal breathlessness responses. These findings reinforce the role of breathlessness as an early marker of disease progression and emphasise the need for early identification and targeted intervention in at-risk populations.

### Caregiver burden due to chronic breathlessness

The burden of chronic breathlessness extends beyond the affected individuals to their informal caregivers. Indeed, those caring for individuals with severe breathlessness (mMRC ≥3) experience significant emotional and physical strain [20^▪▪^]. Caregivers often reported feelings of helplessness, social isolation, and increased stress due to the unpredictable nature of the condition. Factors associated with higher caregiver burden included longer caregiving hours, disruptions in employment, and lack of adequate support from health care providers. These findings highlight the need for including caregiver support in (palliative) care programmes for individuals with chronic breathlessness.

### Societal impact of breathlessness

In a population-based online survey among more than 10 000 Australians, Sunjaya *et al.* found that breathlessness not only has an impact on the individual level, but also imposes a relevant burden on health care systems and the economy [21▪▪]. The authors showed that clinically relevant breathlessness (mMRC grades 2–4) is related to an increase in the use of health care services (consultation of general practitioners and specialists, hospitalisation, medication use, etc.) but also to a reduced likelihood of being employed. Participants reporting mMRC grades 2 or 3 were twice as likely to miss work or school days due to their breathlessness compared to those with an mMRC grade 1. The authors further estimated that the annual societal costs resulting from breathlessness sum up to $12.2 billion in Australia alone when extrapolating the study’s results to the overall population. These costs include direct costs (use of health services) and indirect costs (missed workdays, unemployment). Data on the economic and societal burden of breathlessness are currently scarce, but this study clearly indicates that further investigations of these effects might be important for future research.

## CONCLUSION AND NEEDS FOR FUTURE RESEARCH

Recent advances in research on chronic breathlessness have deepened the understanding of its prevalence, risk factors, and clinical and societal consequences while also proposing new approaches in refining assessment and management strategies. The recognition of chronic breathlessness as a distinct clinical entity in the ICD-11 underscores its significance as a public health concern. Recent epidemiological studies have expanded knowledge beyond high-income countries, revealing substantial variability in prevalence and highlighting the role of socioeconomic and environmental factors in symptom burden. Advances in assessment tools, including exertion-calibrated measures and multidimensional breathlessness scales, have improved the ability to differentiate pathological breathlessness from normal exertional responses, potentially facilitating more precise identification of affected individuals and thereby improving clinical decision-making. However, despite these developments, critical knowledge gaps remain.

Future research should focus on identifying more effective and precise assessment tools and evaluate their applicability in large-population studies. Future investigations should incorporate the current ICD-11 definition of chronic breathlessness as a symptom persisting for more than 8 weeks to differentiate between acute onset and chronicity. Moreover, longitudinal studies are needed to better understand the trajectory of chronic breathlessness and its long-term impact on health outcomes as well as societal impact.
